# Improving Family Physicians’ Vaccine Recommendation Behaviors for Non-Routine Vaccines (Meningococcal, Rotavirus, and HPV): A Two-Phase Intervention Study in Primary Care in Turkey

**DOI:** 10.3390/vaccines14070616

**Published:** 2026-07-14

**Authors:** Özge Kilinç, Ufuk Ünlü

**Affiliations:** 1 Gevaş No. 1 Family Health Center, 65700 Van, Turkey; ozge.kilinc@saglik.gov.tr; 2 Department of Family Medicine, School of Medicine, Tokat Gaziosmanpaşa University, 60100 Tokat, Turkey

**Keywords:** vaccination, family physicians, preventive medicine HPV, rotavirus, meningococcal, continuing professional development, vaccine counseling

## Abstract

Background: In Turkey, meningococcal, rotavirus, and HPV vaccines are not included in the national schedule. We aimed to assess whether a face-to-face educational intervention could improve physicians’ knowledge about these vaccines and influence their subsequent counseling and recommendation practices. Methods: We used a two-phase intervention with pre/post assessment and a 4–6-week follow-up among family physicians. In Phase 1, participants received an in-person structured educational intervention on meningococcal, rotavirus, and HPV vaccines, and their knowledge was evaluated using identical true/false tests administered before and after the session. Phase 2, conducted 4–6 weeks later, measured changes in physicians’ self-reported frequency of providing recommendations about these vaccines, and whether they or their first-degree relatives had received any of the vaccines after the structured educational intervention. Differences in paired proportions were analyzed using McNemar’s test (*p* < 0.05). Results: Ninety-one physicians completed Phase-1; 70 from the same cohort completed Phase-2. Following the educational intervention, correct responses increased across multiple items: e.g., “Rotavirus vaccine is live” 89.0%→100% (*p* = 0.002); “Only humans are reservoir for meningococcal infection” 50.6%→96.7% (*p* < 0.001); “Meningococcal vaccine cannot be co-administered” (false) 70.3%→96.7% (*p* < 0.001). Phase-2 showed more proactive practice: for HPV, 52.9% reported recommending it “upon family request” and 27.1% “at every visit”; for rotavirus, these were 37.1% and 35.7%, respectively. Overall, 52.9% reported having administered one of the three vaccines to themselves or first-degree relatives after structured educational intervention; 62.2% rated the educational intervention’s influence on that decision as 10/10. Conclusions: A structured educational intervention improved family physicians’ knowledge and was associated with more proactive vaccine counseling and recommendation practices.

## 1. Introduction

Vaccination is one of the most effective strategies for protecting human health and preventing infectious diseases, offering both cost-effectiveness and a strong safety profile. Numerous studies have shown that increasing global vaccination rates substantially reduces the burden of preventable diseases [1]. Many countries implement a national vaccination schedule in a systematic manner, guided by the recommendations of international authorities such as the World Health Organization (WHO) [2]. In Turkey, the national immunization program is overseen by the Ministry of Health and currently includes vaccines against hepatitis A and B, poliomyelitis, measles, rubella, mumps, tuberculosis, diphtheria, pertussis, tetanus, and varicella, as well as conjugated pneumococcal and *Haemophilus influenzae* type B infections [3,4].

In addition to these routine vaccines, other licensed vaccines (such as meningococcal, rotavirus, and HPV vaccines) are available in Turkey, although they are not included in the National Immunization Program [5]. Consequently, families must purchase these vaccines out-of-pocket before they can be administered in healthcare facilities. This additional financial burden may constitute an important barrier to vaccine uptake, particularly for families with limited economic resources [6].

Rotavirus is the most common cause of severe acute gastroenteritis in young children [7,8]. Prior to widespread vaccine implementation, rotavirus infection was estimated to cause approximately 440,000 deaths, 2 million hospitalizations, and 25 million outpatient visits annually among children under five years of age worldwide [9]. Rotavirus gastroenteritis remains an important cause of childhood morbidity in Turkey and continues to account for a substantial proportion of acute gastroenteritis cases requiring medical attention. Although rotavirus infections occur year-round, their incidence peaks between September and May in Türkiye [10]. Furthermore, a systematic review including 98 studies conducted in Türkiye reported that rotavirus was detected in 31.8% of children younger than five years presenting with acute gastroenteritis [11]. HPV infection, a sexually transmitted disease, leads to anogenital and oropharyngeal diseases in both men and women. Persistent infection with high-risk HPV genotypes is a major contributor to cervical cancer, with HPV-16 and HPV-18 accounting for approximately 70% of cases globally [12]. According to a regional study conducted in Turkey, HPV-16, the genotype most strongly associated with cervical cancer, was the most frequently detected high-risk HPV genotype, accounting for 29.2% of all high-risk HPV infections [13]. Furthermore, a systematic review of studies conducted in Turkey reported that HPV vaccination uptake ranged from only 0.3% to 6.0%, indicating persistently low vaccine coverage [14]. These findings underscore the substantial unmet need for HPV prevention in Turkey and reinforce the importance of strengthening family physicians’ recommendations for HPV vaccination. Meningococcal disease, particularly meningococcal meningitis, remains one of the most severe infections affecting individuals and communities. *Neisseria meningitidis* commonly affects healthy young individuals and may lead to death within hours of symptom onset [15]. A multicenter study conducted in Türkiye reported a Neisseria meningitidis carriage rate of 7.5% among children and adolescents aged 0–18 years, indicating ongoing circulation of the pathogen [16]. Despite this, a recent study conducted among parents in İstanbul found that only 9.5% of children had received the meningococcal vaccination. Furthermore, only 23.3% of parents reported adequate knowledge regarding meningococcal disease and its prevention through vaccination [17]. These findings highlight the need to improve family physicians’ knowledge and vaccine recommendation practices regarding non-routine childhood vaccines.

Thanks to its well-established primary healthcare system and nationwide family physician network, Türkiye has consistently achieved high vaccination coverage for vaccines included in the National Immunization Program [18]. However, rotavirus, HPV, and meningococcal vaccines are not included in the routine immunization schedule and must therefore be obtained through out-of-pocket payment, creating a substantial financial barrier for many families [6]. Consequently, these vaccines may be perceived as optional rather than essential, leading to an underestimation of the risks associated with vaccine-preventable diseases [17,19]. In this context, primary care plays a central role in vaccine delivery, follow-up, and parental counseling. In Türkiye, family physicians are among the most frequently consulted sources of vaccination-related information [20,21]. Previous studies consistently identify physicians’ recommendations, particularly those of family physicians, as one of the strongest determinants of parental acceptance of non-routine childhood vaccines. Family physicians not only recommend vaccines but also address parents’ concerns, provide evidence-based information on vaccine safety and effectiveness, correct misinformation encountered through the internet and social media, and facilitate informed vaccination decisions [22,23,24]. Despite this pivotal role, studies conducted in Türkiye have shown that many family physicians perceive themselves as inadequately informed or only partially knowledgeable regarding non-routine childhood vaccines [6,25]. These knowledge gaps—particularly regarding vaccine indications, eligibility, administration schedules, co-administration, safety, and effectiveness—may directly influence recommendation practices, as physicians with limited vaccine-related knowledge are significantly less likely to recommend these vaccines to parents [6,26]. Furthermore, heavy workloads and limited consultation times in Turkish primary care settings may hinder discussions about non-routine vaccines, thereby contributing to missed opportunities for vaccination [27]. Given that self-funded childhood vaccines pose similar implementation challenges in many healthcare systems, strengthening family physicians’ knowledge and confidence in counseling parents about non-routine childhood vaccines may contribute to improving vaccine uptake in Turkey while also providing evidence that may inform similar primary care settings where vaccine recommendations rely heavily on family physicians [28].

This interventional study aimed to evaluate the impact of an educational program on family physicians’ knowledge, attitudes, and practices concerning optional childhood vaccinations in the province of Tokat. The intervention consisted of a structured educational intervention on non-scheduled pediatric vaccines, followed by pre-test and post-test assessments to measure changes in knowledge. Additionally, a follow-up survey conducted 4–6 weeks after the intervention evaluated physicians’ counseling practices and their propensity to recommend these vaccines to families. By examining these changes, the study sought to determine whether a targeted educational intervention could improve physicians’ knowledge, information-giving practices, and vaccine recommendation behaviors regarding non-routine childhood vaccines. We anticipate that the findings will contribute meaningful evidence to the literature and support improvements in vaccination counseling within primary care settings.

## 2. Materials and Methods

### 2.1. Study Design

This research was designed as a cross-sectional analytical intervention study evaluating family physicians’ knowledge, attitudes, and behaviors regarding optional childhood vaccinations. The study was conducted between 1 May 2024 and 1 August 2024 in Tokat province.

Tokat is a medium-sized province located in the Central Black Sea region of Turkey, with a mixed urban–rural population. According to the Turkish Statistical Institute, the province has approximately 615,000 inhabitants, of whom approximately 66.3% reside in urban areas and 33.4% in rural areas [29]. The local economy is mainly based on agriculture, livestock farming, public services, and small-scale industry. Primary healthcare services are delivered through Family Health Centers operating under Türkiye’s nationwide family medicine system, which provides universal access to preventive and primary healthcare services. At the time of the study, 211 family physicians were actively working in the province.

### 2.2. Study Population

The study population consisted of all family physicians actively working in the province of Tokat (n = 211). Because the target population was finite and fully identifiable, a total population (census) approach was adopted rather than sampling, and all eligible family physicians were invited to participate. Before enrollment, all eligible physicians were informed about the study objectives and procedures, and written informed consent was obtained from those who agreed to participate. Physicians who agreed to participate were included, whereas those who declined or submitted incomplete questionnaire data were excluded.

### 2.3. Intervention (Educational Program)

The study was conducted in two phases.

In the first phase, a structured educational session titled “Childhood Vaccinations Not Included in the Routine Immunization Program” was delivered in conference halls designated by the Tokat Provincial Directorate of Health. The educational intervention covered the epidemiology and disease burden of meningococcal, rotavirus, and HPV infections; vaccine indications, target age groups, vaccination schedules, co-administration principles, contraindications, vaccine safety, and current recommendations for non-routine childhood vaccines. All sessions were conducted by the same trainer, lasted approximately one hour, and were held in official institutional halls outside routine working hours.

To assess learning outcomes, identical pre-test and post-test questionnaires were administered during the educational session. These agree/disagree questionnaires were developed by the researchers based on a comprehensive review of the relevant literature to assess knowledge related to meningococcal, rotavirus, and HPV vaccines. The questionnaires evaluated participants’ knowledge regarding rotavirus, HPV, and meningococcal vaccines, including disease epidemiology, vaccine indications, target age groups, vaccination schedules, co-administration, contraindications, vaccine safety, and current recommendations. The results of these knowledge assessments are presented separately for each vaccine in Table 1, Table 2 and Table 3. Because the questionnaire was developed specifically for this educational intervention and was intended to assess learning outcomes rather than serve as a standardized measurement instrument, a formal validation study was not performed. Knowledge levels before and immediately after the structured educational intervention were compared to evaluate the short-term effect of the educational intervention.

### 2.4. Data Collection Tools

In the second phase, an online Behavior and Attitude Form—also developed through a literature review—was administered. The second phase was conducted 4–6 weeks after the educational intervention to evaluate short-term knowledge retention and early changes in counseling and vaccine recommendation behaviors, a follow-up interval commonly used in educational intervention studies [30]. To avoid response bias, participants were not informed in advance that a follow-up assessment would be conducted.

The questionnaire examined whether participants’ frequency of informing families about, or recommending, each of the three vaccines had changed. Response options were “no change” or “more frequent.” Those reporting increased frequency were additionally asked whether this occurred:

1. at the family’s request,
2. during every patient visit, or
3. by proactively contacting families via telephone.

Participants were also asked whether they or any of their first-degree relatives had received any of these vaccines following the structured educational intervention. Participants who reported receiving at least one of the study vaccines were asked to rate the influence of the educational intervention on their vaccination decision using a researcher-developed single-item numeric rating scale ranging from 0 (no influence) to 10 (very effective). Responses were summarized descriptively by presenting the number and percentage of participants selecting each score.

### 2.5. Statistical Analysis

Descriptive statistics were used to summarize participants’ baseline characteristics. Categorical variables were presented as frequencies and percentages. Differences between pre-test and post-test scores were evaluated using the McNemar test. A *p*-value of <0.05 was considered statistically significant. All analyses were performed using IBM SPSS Statistics version 20 (SPSS Inc., IBM Company, Somers, NY, USA).

## 3. Results

Of the 211 eligible family physicians, 91 (43.1%) participated in the first phase of the study. Of these, 70 (76.9%) completed the second-phase follow-up questionnaire.

Regarding rotavirus-related items, complete improvement was observed in the post-test for several questions. All participants correctly identified that the rotavirus vaccine is a live vaccine, that rotavirus is the leading cause of hospitalization due to gastroenteritis, and that there is no specific treatment for rotavirus gastroenteritis, despite incorrect responses being present in the pre-test. Substantial improvements were also noted in knowledge about the management of vomiting following rotavirus vaccination and the availability of multiple rotavirus vaccine types in Turkey. The percentages of correct and incorrect responses are presented in Table 1.

HPV-related items also demonstrated significant improvement. Post-test accuracy rates were markedly higher than pre-test rates across all questions, as shown in Table 2.

The greatest improvement was observed in items related to meningococcal infections. All questions showed statistically significant differences (*p* < 0.001), and in the post-test, all participants responded correctly to both meningococcal-specific knowledge items. Percentages of correct and incorrect responses are shown in Table 3.

In the second phase of the study, changes in physicians’ vaccine recommendation behaviors were evaluated. After the structured educational intervention, 51.4% of physicians reported more frequently recommending the rotavirus vaccine, 37.1% reported increased recommendations for HPV, and 41.4% for meningococcal vaccines—either during visits or via phone contact. In response to whether they had received any of the vaccines themselves or administered them to first-degree relatives after the structured educational intervention, 37 physicians answered yes, while 33 answered no. Among these vaccines, the rotavirus vaccine was the most frequently administered.

The evaluation of the perceived impact of the structured educational intervention showed that the study’s primary aim was achieved: 94.6% of participants reported that the structured educational intervention influenced their vaccination-related decisions. The variables examined in the second phase are summarized in Table 4.

## 4. Discussion

Our study showed that a brief face-to-face structured educational intervention for family physicians significantly increased knowledge about rotavirus, HPV, and meningococcal vaccines, and also resulted in a significant improvement in information-giving and recommendation behaviors at 4–6 weeks follow-up. Improvements were also observed in frequently confused areas such as “included in the national schedule” and “cannot be administered with other vaccines” (all *p* < 0.001). At the behavioral level, the majority of physicians reported making more frequent recommendations to families after the structured educational intervention. Furthermore, 52.9% of physicians reported administering a specific vaccine to themselves or a first-degree relative, and 62.2% reported that the structured educational intervention had a “10/10” impact on their decision, suggesting that the structured educational intervention was effectively translated into practical action.

The high and increasing accuracy rates observed in our study are consistent with findings reported both nationally and internationally. In a study conducted in Nigeria, 85.7% of healthcare workers identified rotavirus as the leading cause [31]. In our sample, the accuracy rate for this question increased from 84.6% in the pretest to 100% after structured educational intervention. We believe this improvement demonstrates that fundamental epidemiological knowledge in primary care can be effectively reinforced through structured educational interventions.

Knowledge regarding the vaccination schedule—such as dose intervals and appropriate steps following post-vaccination vomiting—also increased significantly after the structured educational intervention. In Turkey, knowledge of the “interval of ≥4 weeks between doses” was reported as 75.47% [32]. In our study, the correct response rate for this item rose from 83.5% to 96.7%, indicating that, while pre-intervention knowledge was consistent with previous reports, the educational intervention substantially improved physicians’ knowledge of appropriate vaccination timing.

The item concerning “the earliest age at which the first dose can be administered” was the most challenging in the pretest (36.3%). Although following the educational intervention, accuracy improved, the increase was less pronounced compared with other items. The substantial improvement observed in our study regarding knowledge of the available rotavirus vaccine types (Rotarix and RotaTeq), from 58.2% to 98.9%, is consistent with previous Turkish studies reporting that 82–85% of family physicians correctly identified the two licensed rotavirus vaccines [32].

The item stating that “the HPV vaccine can be administered to both girls and boys” showed one of the strongest improvements in knowledge following the structured educational intervention, increasing from 71.43% to 98.9%. Knowledge gaps and hesitations regarding the recommendation of HPV vaccination for boys in primary care have been well documented in the literature. In Anderson et al., only 20% of participants correctly identified that the vaccine is effective and recommended for boys [33]. Similarly, Duval et al. reported that 93.6% of family physicians primarily associated HPV vaccination with “girls before sexual activity” [34]. Evidence from Turkey reflects comparable concerns; one study found that knowledge regarding the applicability of the vaccine to both boys and girls aged 9–18 was only 42.5% [35]. Our findings are also consistent with another Turkish educational study conducted among family health workers, which demonstrated significant improvements in knowledge and attitudes toward HPV infection, HPV vaccination, and vaccination of both girls and boys following the educational intervention. Together, these findings suggest that structured educational interventions can effectively address knowledge gaps regarding HPV vaccination among primary healthcare professionals in Türkiye [36]. In this context, the substantial improvement observed in our study suggests that misconceptions related to HPV vaccination in boys can be effectively corrected through brief, targeted educational interventions.

The findings related to meningococcal disease demonstrated substantial and consistent improvements following the structured educational intervention. The proportion of participants correctly identifying that the meningococcal vaccine is included in the national immunization schedule increased from 63.7% to 85.7% (*p* < 0.001), although 14.3% still responded incorrectly. In comparison, Tanrıkulu et al. reported a correct response rate of 91.2% among 102 participants in Adana [37]. While our post-intervention improvement is notable, the residual error highlights the need for continuous updates in medical knowledge, especially for family physicians who are most frequently responsible for vaccine administration.

Knowledge regarding disease burden also improved markedly. Correct responses to the item stating that 70% of meningococcal deaths occur in children under five increased from 78.0% to 100% (*p* < 0.001). Similarly, knowledge that meningococcal disease is most common in children under one year of age increased significantly from 67.0% to 98.9% (*p* < 0.001). Tanrıkulu et al. noted that in developing countries, meningococcal infection is most frequent in children under two, whereas in developed countries it occurs more commonly after age ten, with a correct response rate of 76.5% [37]. These findings suggest that our results align with existing evidence.

Substantial gains were also observed in understanding transmission and coadministration principles. Correct responses to “humans are the only reservoir for meningococcal infection” increased from 50.6% to 96.7% (*p* < 0.001), whereas Tanrıkulu et al. reported 85% correct responses [37]. Knowledge of the safe coadministration of meningococcal vaccines with other vaccines is particularly important given the crowded childhood immunization schedule and the availability of non-routine vaccines. Knowledge of meningococcal vaccine availability improved significantly. While 76.9% correctly stated in the pretest that only a single type of meningococcal vaccine is available in Turkey, this increased to 100% after structured educational intervention (*p* < 0.001). Achieving full accuracy across all participants further supports the effectiveness of the educational intervention and suggests that periodic educational activities may help maintain up-to-date vaccine knowledge among primary care physicians. Overall, statistically significant improvements were observed across all meningococcal-related items, demonstrating that structured educational intervention can successfully address knowledge gaps and enhance readiness for vaccine-preventable disease management in primary care settings.

In the 4–6 weeks following the structured educational intervention, physicians reported an overall increase in the provision of information and recommendations to families regarding all three vaccines. Counseling patterns indicated that the most common approach remained “upon family request,” followed by “at every visit.” For the rotavirus vaccine, information was provided upon family request in 45.7% of cases, at every visit in 28.6%, via telephone consultation in 10.0%, and rarely or never in 15.7%. The corresponding proportions for the meningococcal vaccine were 14.3%, 48.6%, 28.6%, and 8.6%, respectively, while for the HPV vaccine they were 11.4%, 45.7%, 27.1%, and 15.7%, respectively. Regarding vaccine recommendations, the distribution patterns were 11.4%, 37.1%, 35.7%, and 15.7% for rotavirus; 14.3%, 44.3%, 30.0%, and 11.4% for meningococcal; and 10.0%, 52.9%, 27.1%, and 10.0% for HPV. Overall, these findings suggest that proactive counseling at every visit has gained prominence following the structured educational intervention; however, a demand-driven approach remains the predominant practice across all vaccine types.

This behavioral improvement aligns with physicians’ self-reported impact of the structured educational intervention and personal vaccination practices: 52.9% reported administering at least one vaccine to themselves or an immediate family member, and 62.2% rated the structured educational intervention as having a “10/10” influence on their decision-making. Similarly, a Turkish study involving pediatricians and family physicians reported that nearly half of the participants (49%) perceived themselves as having insufficient knowledge regarding non-routine childhood vaccines. Physicians who considered themselves adequately informed were significantly more likely to provide vaccine-related information and recommendations to families than those who reported insufficient knowledge (*p* < 0.001) [26]. These observations are further supported by a nationwide Turkish study involving family physicians and family health personnel, which identified insufficient vaccine-related knowledge as one of the principal barriers to recommending rotavirus, HPV, and meningococcal vaccines. In addition to inadequate knowledge, vaccine cost and concerns about adverse effects were also reported as important factors limiting vaccine recommendations. The authors emphasized that educational programs aimed at improving healthcare professionals’ knowledge and confidence are essential for strengthening vaccine recommendation practices [25]. Taken together, these findings support our results, demonstrating that even a brief educational intervention may translate into more proactive counseling and vaccine recommendation behaviors in primary care.

The broader implications of these findings should be considered within the central role of family physicians in vaccination delivery and preventive healthcare. Family physicians are often the first point of contact for parents seeking vaccination-related information and play a key role in addressing concerns, countering misinformation, and facilitating informed decision-making [21]. Previous research has consistently identified physician recommendation as one of the strongest predictors of vaccine acceptance, particularly for vaccines outside routine immunization schedules [17,38]. Therefore, interventions that strengthen physicians’ vaccine-related knowledge and confidence may contribute not only to improved recommendation practices but also to more effective implementation of vaccination strategies in primary care settings [39,40].

In the Turkish context, the administration of vaccines not included in the national immunization schedule—such as HPV, rotavirus, and meningococcal vaccines—largely depends on physician guidance and family initiative [6]. Our findings suggest that structured educational intervention can quickly address these knowledge gaps, as evidenced by the increased proportion of “information/advice at every visit” after the program. Moreover, the substantial use of telephone counseling (e.g., HPV information provided at 15.7%) indicates that remote communication channels are being utilized more frequently, which may help overcome access barriers for vaccines requiring specialized procurement.

The strength of this study is that the intervention focused directly on practical issues (dose intervals, co-administration, post-vomiting approach, etc.) and captured its effects on behavior with a short follow-up. This study was conducted as an intervention study in which HPV, meningococcal, and rotavirus vaccines were evaluated together, and knowledge, attitudes, and recommendation practices were assessed using a pretest–post-test design. However, demographic characteristics of the participating physicians were not collected, as the study was designed to evaluate the overall effectiveness of the educational intervention rather than differences between participant subgroups. Consequently, subgroup analyses according to physician characteristics could not be performed. In addition, the inclusion of physicians from a single province and the use of self-reported behavioral measures may limit the generalizability and precision of the findings. Furthermore, because the follow-up period in the second phase was limited to 4–6 weeks, no conclusions can be drawn regarding the long-term sustainability of the observed effects.

## 5. Conclusions

In conclusion, this study demonstrated that a structured educational intervention significantly improved family physicians’ knowledge regarding rotavirus, HPV, and meningococcal vaccines, which are not included in the Turkish National Immunization Program. Improvements were also observed in physicians’ self-reported counseling and vaccine recommendation practices during short-term follow-up, suggesting that enhanced knowledge may positively influence preventive healthcare delivery in primary care. These findings indicate that a structured educational intervention can improve physicians’ knowledge and may positively influence their counseling and vaccine recommendation practices regarding non-routine childhood vaccines. Future multicenter studies with longer follow-up are warranted to determine whether these improvements are sustained over time and are associated with increased uptake of non-routine childhood vaccines.

## Figures and Tables

**Table 1:** Percentages of correct and incorrect answers to questions regarding rotavirus infection.

Question	Pretest		Post-Test		*p*
	Agree	Disagree	Agree	Disagree	
The rotavirus vaccine is a live vaccine.	89% *	11%	100% *	0	0.002
The most common cause of gastroenteritis requiring hospitalization is rotavirus gastroenteritis.	84.6% *	15.4%	100% *	0	<0.001
There is no specific treatment for rotavirus gastroenteritis.	91.2% *	8.8%	100% *	0	0.008
There should be at least a 4-week interval between rotavirus vaccine doses.	83.5% *	16.5%	96.7% *	3.3%	0.002
If the child vomits within 10 min after rotavirus vaccination, the vaccine should be repeated.	52.7%	47.3%*	4.4%	95.6% *	<0.001
The earliest time the rotavirus vaccine can be administered is when the baby is 4 weeks old.	36.3% *	63.7%	51.7% *	48.3%	0.003
There is only one type of rotavirus vaccine in our country.	41.8%	58.2% *	1.1%	98.9% *	<0.001
Rotavirus vaccine is not administered to children with immune deficiency or suspected immunodeficiency.	69.2% *	30.8%	85.7% *	14.3%	0.001

* defines the correct answer.

**Table 2:** Percentages of correct and incorrect answers to questions regarding HPV infection.

Question	Pretest		Post-Test		*p*
	Agree	Disagree	Agree	Disagree	
HPV vaccine can be administered to both girls and boys.	71.4% *	28.6%	98.9% *	1.1%	<0.001
When HPV vaccines are administered between the ages of 9 and 14, a higher rate of antibody response occurs compared to older ages.	86.8% *	13.2%	98.9% *	1.1%	0.001
HPV vaccine is recommended in two doses for girls aged 9–14 and three doses for those aged 15 and older.	80.2% *	19.8%	96.7% *	3.3%	<0.001

* defines the correct answer.

**Table 3:** Percentages of correct and incorrect answers to questions regarding meningococcal infections.

Question	Pretest		Post-Test		*p*
	Agree	Disagree	Agree	Disagree	
Most meningococcal diseases occur in healthy individuals with no previously identified risk factors.	56% *	44%	89% *	11	<0.001
Meningococcal vaccine is included in the national vaccination programme.	36.3%	63.7% *	14.3%	85.7% *	<0.001
70% of deaths from meningococcal infection occur in children under 5 years of age.	78% *	22%	100% *	0	<0.001
Meningococcal infections are most commonly seen in children under 1 year of age in our country.	67% *	33%	98.9% *	1.1%	<0.001
Humans are the only reservoir for meningococcal infections.	50.5% *	49.5%	96.7% *	3.3%	<0.001
There is only one type of meningococcal vaccine in our country.	23.1%	76.9% *	0	100% *	<0.001
Meningococcal vaccine cannot be administered with other childhood vaccines.	29.7%	70.3% *	3.3%	96.7% *	<0.001

* defines the correct answer.

**Table 4:** Distribution of variables examined in the second phase.

Variables	Status	n	%
Recommending Rotavirus vaccination after the educational intervention	not changed	26	37.1
	as the family requests information	8	11.4
	during all visits	25	35.7
	via phone contact	11	15.7
Recommending Meningococcal vaccines after the educational intervention	not changed	31	44.3
	as the family requests information	10	14.3
	during all visits	21	30.0
	via phone contact	8	11.4
Recommending HPV vaccines after the educational intervention	not changed	37	52.9
	as the family requests information	7	10.0
	during all visits	19	27.1
	via phone contact	7	10.0
Have you ever had any of these vaccines administered to yourself or your first-degree relatives after the educational intervention?	Yes	37	52.9
	No	33	47.1
If your answer to the above question is yes, please select the vaccine you have applied	HPV	15	40.5
	Meningococcal	6	16.2
	Rotavirus	16	43.2
If you have been vaccinated, please rate the impact of the education on your decision between 0 and 10. (0: no effect/10: very effective)	0 point	2	5.4
	5 points	1	2.7
	7 points	3	8.1
	8 points	5	13.5
	9 points	3	8.1
	10 points	23	62.2

## Data Availability

This study is based on the medical specialization thesis of Özge Kılınç. The datasets used and/or analyzed during the current study are available from the corresponding author on reasonable request.
